# Short-Chain Fatty Acids Modulate Healthy Gut Microbiota Composition and Functional Potential

**DOI:** 10.1007/s00284-022-02825-5

**Published:** 2022-03-14

**Authors:** Christine Tara Peterson, Josue Perez Santiago, Stanislav N. Iablokov, Deepak Chopra, Dmitry A. Rodionov, Scott N. Peterson

**Affiliations:** 1 Department of Family Medicine, Center of Excellence for Research and Training in Integrative Health, School of Medicine, University of California San Diego, 9500 Gilman Drive #0725, La Jolla, CA USA; 2 Puerto Rico Omic Center Genomics Core Division of Cancer Biology, University of Puerto Rico Comprehensive Cancer Center, San Juan, Puerto Rico; 3 Russian Academy of Sciences, Institute for Information Transmission Problems, Moscow, Russia; 4 Bioinformatics and Structural Biology Program, Sanford Burnham Prebys Medical Discovery Institute, La Jolla, CA USA; 5 Tumor Microenvironment and Cancer Immunology Program, Sanford Burnham Prebys Medical Discovery Institute, La Jolla, CA USA

## Abstract

Many studies have focused on the metabolic capacity of human gut microbiota to produce short-chain fatty acids and subsequent effects on host physiology. Given scarce data on how SCFAs produced by gut bacteria participate in cross-feeding to influence community structure and function, we evaluated the potential of SCFAs to modulate human gut microbiota in vitro. We employed anaerobic fecal cultivation in chemically defined medium supplemented with one of nine SCFAs to determine effects on both gut microbial community structure via 16S rRNA sequencing and function via genome reconstruction analysis. Each SCFA displayed significant and unique modulatory potential with respect to the relative abundance of bacterial taxa. Analysis of SCFA-supplemented communities revealed that alterations of individual closely related phylotypes displayed coherent changes, although exceptions were also observed which suggest strain-dependent differences in SCFA-induced changes. We used genome reconstruction to evaluate the functional implications of SCFA-mediated restructuring of fecal communities. We note that some SCFA-supplemented cultures displayed a reduction in the predicted abundance of SCFA producers, which suggests a possible undefined negative feedback mechanism. We conclude that SCFAs are not simply end-products of metabolism but also serve to modulate the gut microbiota through cross-feeding that alters the fitness of specified taxa. These results are important in the identification of prebiotics that elevate specific SCFAs for therapeutic benefit and highlight SCFA consumers as a salient part of the overall metabolic flux pertaining to bacterial fermentative processes.

**Supplementary Information:**

The online version contains supplementary material available at 10.1007/s00284-022-02825-5.

## Introduction

One of the central roles of the gut microbiota is to serve as an intermediary between diet and the physiology and health of the host. A well-studied example is the role of gut microbes in the harvest of energy from otherwise indigestible fibers derived from plants, such as vegetables and fruits. The catabolism of dietary fiber is carried out by taxonomically distinct groups of bacteria (e.g., *Bifidobacterium*,* Bacteroides*,* Parabacteroides*) that encode large suites of glycosyl hydrolases that liberate mono-, di-, and oligo-saccharides from dietary glycans. The cooperative activities of microbes ensure efficient breakdown of varied sugar polymers. Many glycosyl hydrolases are localized on the microbial cell surface which is thought to provide a mechanism of cross-feeding that provides liberated sugars to other bystander saccharolytic microbes and the host. It is estimated that about 10% of the human’s daily calories are derived from this process [1].

Sugar fermentation is a property shared by the majority of gut microbes. Under anaerobic conditions present in the colon, these sugars are metabolized by a variety of pathways for energy (i.e., ATP) leading to the generation of SCFAs, such as formate, acetate, propionate, butyrate, succinate, and lactate. Fermentation of branched chain amino acids leads to the production of additional SCFAs, such as isobutyrate, isovalerate, and others. SCFAs represent end-products of metabolism for many bacteria but may represent potential substrates for others thus defining a form of cross-feeding employed by the gut microbiota. The high density of microbes residing in the distal colon (~10^12^/gram) and functional redundancy with respect to some of the metabolites these bacteria produce enables some microbial metabolites to reach physiologically relevant levels and produce a significant impact on the host.

One of the best studied syntrophic networks operating in gut microbiota–host interactions pertains to processes involved in energy extraction from dietary glycans, sugar fermentation, and production of short-chain fatty acids [2]. When cultured with resistant starch 2 (RS2), *Ruminococcus bromii* generated significant quantities of reducing sugars but displayed a poor capacity for utilization of carbohydrate substrates. When *R. bromii* was co-cultured with *B. thetaiotaomicron*, the total carbohydrate utilization was increased [3]. *Ruminococcus bromii* generates formate, acetate, and ethanol when provided resistant starch. When co-cultured with *Blautia hydrogenotrophica*, formate was consumed and additional acetate was produced [4]. A consequence of sugar fermentation is the generation of H_2_. Fermentation efficiency is reduced as H_2_ accumulates, inhibiting bacterial NADH-dependent dehydrogenases. Mutualism between *B. thetaiotaomicron* and *D. piger* has been reported [5]. In co-colonized mice, *B. thetaiotaomicron* increases the fitness of *D. piger* by providing sulfate, compensating for the lack of sulfatase functions encoded by the *D. piger* genome. *D. piger* consumes H_2_ thus allowing fermentation efficiency of *B. thetaiotaomicron* to be maintained. Moreover, the activities of some species alter the metabolic preferences of others. *Methanobrevibacter smithii* directs *B. thetaiotaomicron* to preferentially ferment dietary fructans that generate acetate and formate that are subsequently consumed by *M. smithii* for methanogenesis [6]. Mice co-colonization with *M. smithii* and *B. thetaiotaomicron* promoted increases in cell growth of both species, indicating a mutualistic interaction. In addition, *Bifidobacterium* generate lactate through metabolism of various prebiotic substrates such as ITF and AXOS that cross-feeds a variety of microbes that convert lactate to butyrate [7].

The growing appreciation of the number of syntrophic relationships engendered by the gut microbiota motivated the current study to evaluate the extent that SCFAs may serve as substrates for microbes and to determine how this affects the fitness of taxa as measured by relative abundance. Currently, little is known about how SCFAs produced by gut bacteria participate in cross-feeding to influence community structure and function. We hypothesized that the SCFA substrates may be potent drivers that alter the gut microbiota composition thereby redirecting community metabolism.

Here, we report the substantial modulation of fecal microbiota composition in a chemically defined medium supplemented with a single-SCFA substrate. Both increased and decreased microbial relative abundances were observed for each of the 9 SCFAs tested which include formate, acetate, propionate, butyrate, lactate, succinate, valerate, isobutyrate, and isovalerate. These effects were largely strain-dependent and rarely impacted all microbes belonging to species groups uniformly, although strong species-level trends were observed. Taxa altered by one SCFA were often also responsive to several others tested which suggests that some taxa are more sensitive to SCFAs than others. We assessed the functional implications of SCFA-mediated restructuring of microbial communities focusing on SCFA production, amino acid biosynthesis and degradation and vitamin biosynthesis. While community-based metabolic phenotype profiling of these effects demonstrates the strong modulatory capacity of SCFAs on community structure, detailed analysis to reveal the mechanisms associated with these changes will require co-culture experiments that will assess substrate utilization and potential inhibitory action of SCFAs.

## Methods

### Study Participants and Sample Collection

Healthy women and men aged 30–60 years that had previously adhered to a vegetarian or vegan diet for >1 year were recruited to donate a single-stool sample. This study was carried out in accordance with the recommendations of Sanford Burnham Prebys Medical Discovery Institute Institutional Review Board (IRB-2014-020) and guidelines with written informed consent from all subjects. All subjects gave written informed consent in accordance with the Declaration of Helsinki. The protocol was approved by the Sanford Burnham Prebys Medical Discovery Institute Institutional Review Board. Participants ate their normal diets and donated a morning fecal sample in stool hats (Fisher Scientific). The fecal samples were transferred to conical tubes and stored at −80 °C until subsequent processing.

### SCFAs Examined in the Current Microbiome Study

We examined 9 SCFAs which include formate, acetate, propionate, butyrate, d-lactate, valerate, succinate, isobutyrate, and isovalerate (Sigma).

### Anaerobic Fecal Cultures

A 2× concentrate of chemically defined medium (CDM) containing 100 mM HEPES, 4.4 mM KH_2_PO_4_, 20 mM Na_2_HPO_4_, 120 mM NaHCO_3_, 8 mM of each amino acid except leucine (30 mM), 20 mL ATCC, Trace Mineral Supplement, nucleoside bases (200 mg/L), inosine, xanthine, adenine, guanine, cytosine, thymidine and uracil (800 mg/L), choline (200 mg/L), ascorbic acid (1 g/L), lipoic acid (4 mg/L), hemin (2.4 mg/L), and myo-inositol (800 mg/L). Resazurin (2 mg/L) was added to visually monitor dissolved oxygen. The pH of the media was adjusted to 7.4. CDM contains amino acids but no exogenous carbohydrate. It should be noted that amino acid degradation pathways can generate SCFAs. Despite this complication, all cultures analyzed differed only by the supplementation of a single SCFA, therefore the effect of these additions allow the modulatory effects of SCFAs on fecal communities to be identified, although distinguishing primary vs secondary effects of SCFA supplementation is confounded.

CDM (2×) without SCFAs was reduced in an anaerobic chamber (Coy Labs) for 2 days together with several aliquots of H_2_O (4 mL). To ensure equal inoculums across cultures frozen stool sample generated from an equal volume of stool pooled from 12 healthy vegetarian participants were used to inoculate 2× CDM that was then distributed (1 mL) into individual tubes. Individual SCFAs were introduced to reduce H_2_O to a final concentration of 20 mM. One mL of SCFA solution was added to 2× CDM to generate cultures containing approximately 1 × 10^6^ cells in 1× CDM with or without 10 mM SCFA. Due to the strong buffering capacity of the media, the addition of SCFAs to media had nominal effects on pH, reducing it to 7.2–7.3, therefore no further adjustments were made prior to fecal inoculation. Cultures (9% H_2_, 81% N_2_) were grown statically for 2 days at 37 °C as technical replicates (*n* = 4) and grown to approximate saturation.

### Microbial DNA Isolation

Genomic DNA was isolated from cultures using the procedures of the QiaAmp DNA stool kit (Qiagen) with a modification that included bead beating with the Thermo FastPrep instrument (MP Bio) to ensure uniform lysis of bacterial cells.

### 16S rRNA Sequence Analysis

Multiplexed 16S rDNA libraries were prepared using standard 16S metagenomic sequencing library protocols from Illumina, which uses V3–V4 region of 16S rDNA for target amplification, and subsequent analysis was performed in the CLC Microbial Genomics Module 2.5 (Qiagen) and R [8] as we have described previously [9–11]. Output from CLC was used for phylotype-based analyses (Supplementary Table S2). We used Qiime 2 for all other taxonomic analyses at the species level and higher and for subsequent genome reconstruction. Briefly, raw sequence reads were filtered, denoised, paired-read merged, and chimeras removed using the default parameters in dada2 [12] to generate an abundance table with amplicon sequence variants (ASVs) representing individual 16S sequences. To assign taxonomic descriptions to the obtained ASVs we used the multi-taxonomy approach (MTA). Each ASV sequence was aligned with 16S sequences from Ribosomal Database Project (RDP, version 11.5). The obtained alignments were sorted by the percent identity with maximum values denoted as M. We further collected and processed taxonomic assignments for identified 16S rRNA sequences with identities higher than the M‒(1‒M)/4 threshold. Resulting multi-taxonomy assignments consisted of one or more taxonomic names separated by “/.” To account for variable 16S rRNA gene copy number in reference genomes, we further renormalized each sample’s ASV abundance by average 16S copy numbers at each taxonomic level provided by the rrnDB database [13].

### Genome Reconstruction of Metabolic Pathways and Calculation of Metabolic Phenotype Profiles

To predict metabolic potential of microbial taxa identified by 16S rRNA analysis we utilized the Phenotype Profiler tool (PhenoBiome Inc., San Francisco, CA). To predict metabolic capabilities of 2856 reference genomes representing 690 microbial species from human gut, we used a subsystem-based approach implemented in microbial community SEED (mcSEED) platform [14], as we have described previously [10, 15, 16]. Each reference genome in each analyzed metabolic subsystem was assigned a binary (“1” or “0”) phenotype reflecting the presence/absence of a complete amino acid/vitamin or SCFA synthesis pathway. The obtained binary phenotype matrix (BPM) for metabolic phenotype distributions in the reference genomes was used to calculate a probabilistic estimate *P* for each mapped taxa obtained from 16S analysis to possess a certain binary metabolic phenotype as previously described [15]. Community Phenotype Index (CPI) values for each 16S sample were calculated as a sum of respective *p* values of each taxa multiplied by their relative abundance. CPI provides a fractional representation of cells in the community possessing a specific metabolic pathway (on the scale 0–100%).

### Statistical Analyses

Differences in the Shannon alpha diversity measurements, the relative abundance of particular gut microbial taxa, community phenotypes, including SCFA production, vitamin biosynthesis, amino acid biosynthesis, and degradation pathways in experimental compared to control cultures was assessed using a double-tailed Mann–Whitney test. Results of all statistical analyses are provided (Supplementary Table 1).

### Data Availability

All 16S rRNA sequences have been deposited at NCBI SRA database and can be found at https://www.ncbi.nlm.nih.gov/genbank/. The Bioproject ID is PRJNA725898 and accession numbers are SAMN18914736–SAMN18914775.

## Results

We profiled the microbiota of a pool consisting of 12 healthy vegetarian stool samples in a chemically defined medium (CDM) supplemented with a single SCFA including formate, acetate, propionate, butyrate, lactate, succinate, valerate, isobutyrate, and isovalerate to assess whether they differentially impacted the fitness of microbes within these communities. The CDM included amino acids but did not provide any carbohydrate sources for SCFA production. We amplified the V3–V4 region of the 16S rDNA gene from replicate cultures and the resulting sequences derived from control cultures (CDM) were compared to cultures supplemented with one of nine tested SCFAs.

### SCFAs Modulate the Relative Abundance of Numerous Taxa

Sequence data were analyzed at the phylotype level, using CLC, wherein sequences differing by as few as 1 base were maintained separately and no merging of unique sequences was performed. Unique sequences were used to conduct BLAST to identify the most closely related named species (average % identity = 98.6%). In total, we enumerated 457 unique phylotypes that defined 125 bacterial species groups (Supplementary Table S2). We observed 337 phylotypes in control cultures.

**Fig. 1:**
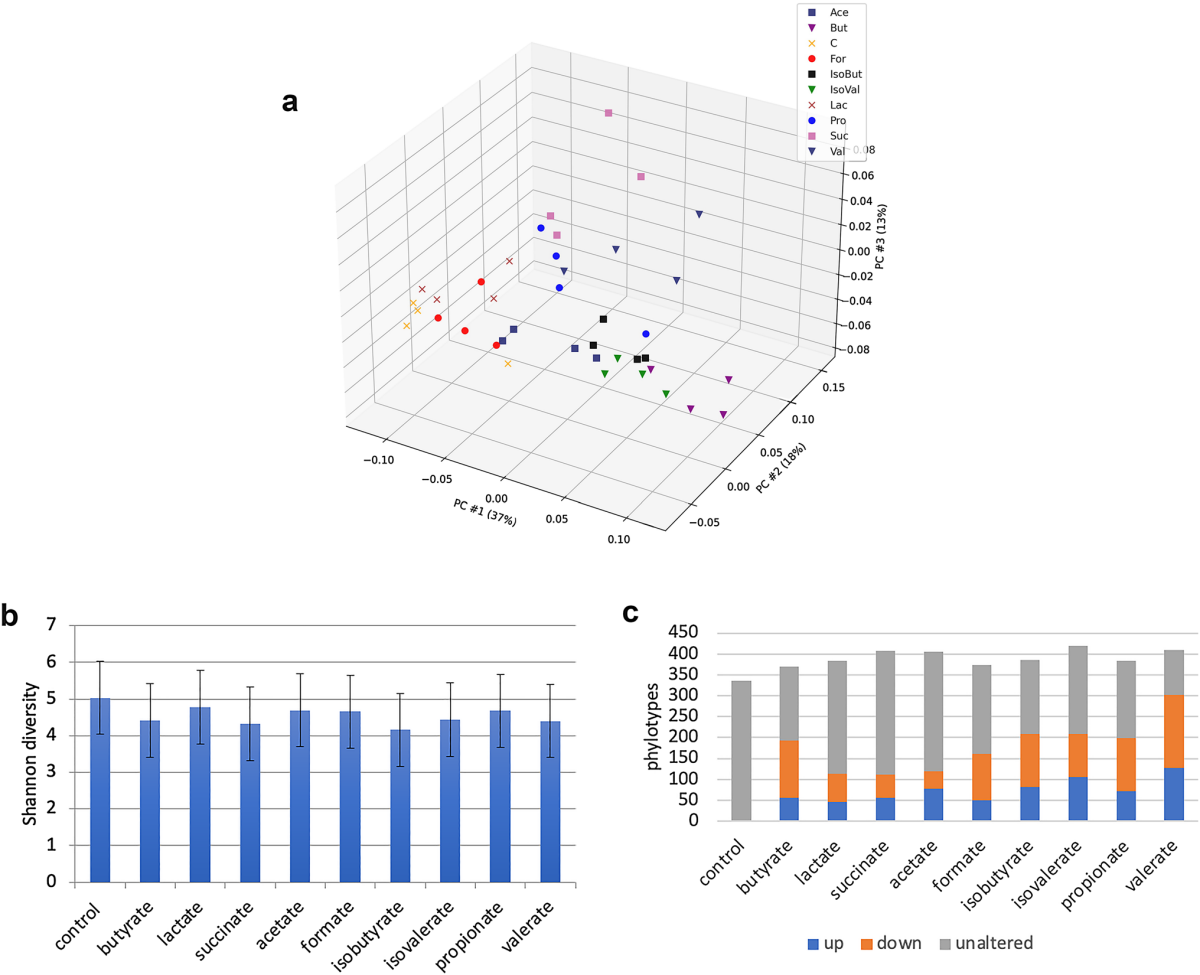
Principal component analysis of SCFA-supplemented cultures. **a** Bray–Curtis β diversity measures of control cultures compared to those supplemented with SCFAs. **b** Alpha diversity. Shannon diversity measures for control cultures compared to those observed in CDM supplemented with a single SCFA. **c** Modulatory effects of SCFAs. The average relative abundance of individual taxa in SCFA-supplemented cultures was compared to control cultures. Taxa displaying increased or decreased relative abundance (>fivefold) and the sum of these groups were summed and reported as altered. Taxa displaying changes < fivefold relative to control cultures were summed and reported as unaltered

We calculated β diversity using Bray–Curtis distances of control and SCFA-supplemented communities as determined by Qiime 2 analysis pipeline to assess global changes in microbial community composition (Fig. 1a). The resulting plot shows that most SCFA-supplemented cultures were distinct from that of control cultures. Cultures supplemented with lactate and formate were the least altered, whereas all other cultures clustered separately. Cultures supplemented with isovalerate, isobutyrate, and to a lesser extent butyrate clustered together, whereas other SCFAs selected for unique communities. The supplementation of media with SCFA did not significantly alter the alpha diversity of cultures compared to controls (Fig. 1b). However, it is notable that the number of phylotypes observed in all SCFA-supplemented cultures was higher compared to controls (Fig. 1c). Cultures supplemented with valerate, isovalerate, succinate, and acetate displayed the highest number of phylotypes. Compared to control cultures that provide only amino acids as a potential energy source, each SCFA-supplemented culture displayed a substantial modulation of phylotypes, including both increased and decreased relative abundance (Fig. 1c). Among the changes in relative abundance observed, most SCFAs displayed a larger number of phylotypes with reduced relative abundance, with the exception of succinate and lactate. Lactate, succinate, and acetate-supplemented cultures displayed the lowest modulatory capacity. Valerate and isovalerate increased the relative abundance of the largest number of phylotypes, whereas butyrate, formate, and lactate increased the relative abundance of the fewest. Isobutyrate, propionate, butyrate, and valerate-supplemented cultures displayed the largest effects in terms of the number of phylotypes displaying reduced relative abundance.

### Taxonomic Analysis of SCFA Impact on Microbiota Community Structure

**Fig. 2:**
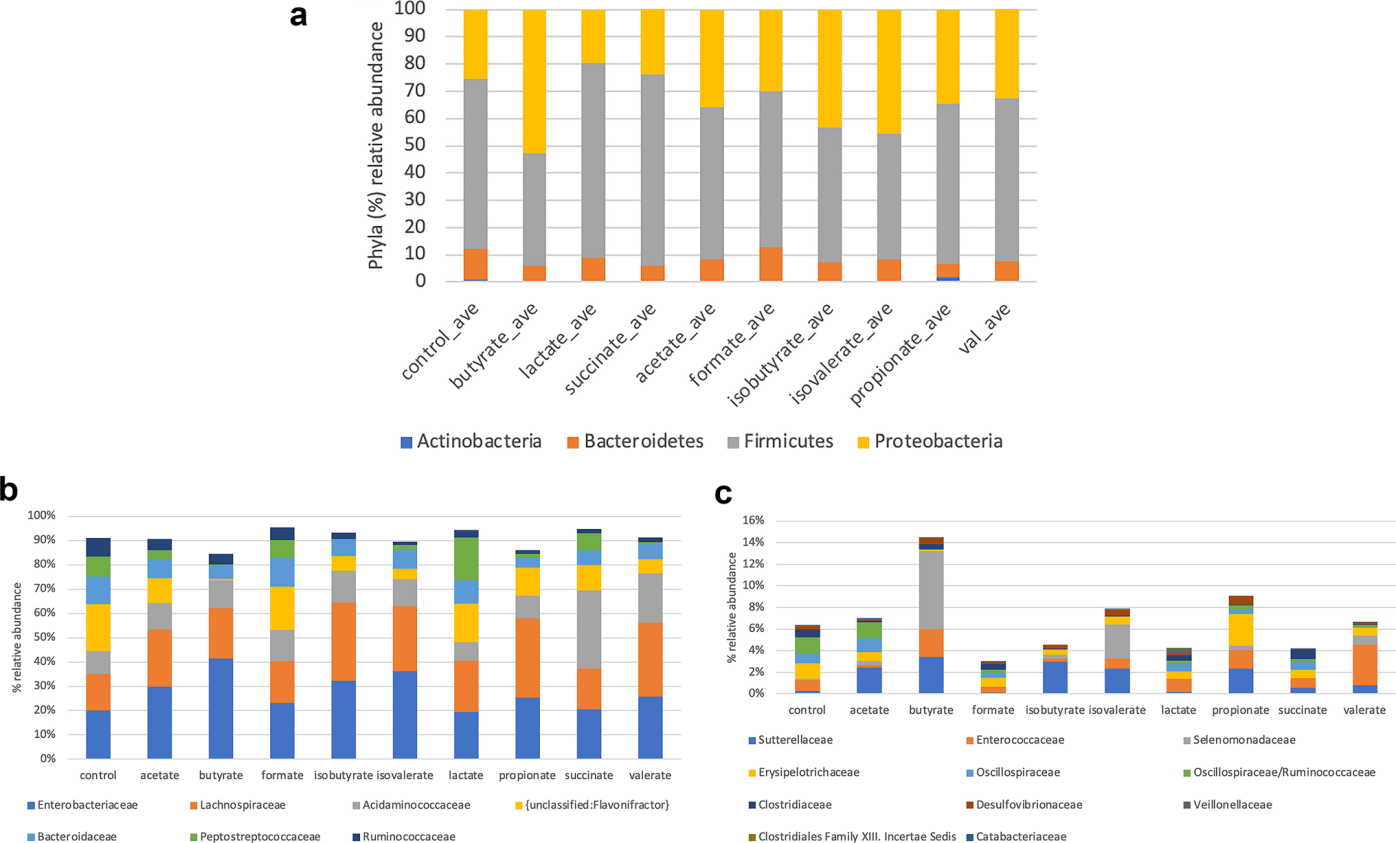
Alterations at the phylum and family level by SCFAs. **a** The average relative abundance of individual taxa belonging to one of four phyla was summed for each SCFA-supplemented cultures and compared control cultures. **b** Alterations in dominant families by SCFAs. **c** Alterations in families of lower abundance by SCFAs

Analysis of community profiles at the phylum level revealed that cultures were dominated by 4 phyla, namely Firmicutes, Bacteroidetes, Proteobacteria, and to a lesser extent Actinobacteria (Fig. 2a). Statistically significant alterations are shown in (Supplementary Table S1). Compared to control cultures, butyrate-supplemented cultures reduced the relative abundance of Firmicutes (*P* = 0.03) and Bacteroidetes (*P* = 0.03) but increased the relative abundance of Proteobacteria (*P* = 0.03). The relative abundance of Proteobacteria was increased by several additional SCFAs including acetate, propionate, isobutyrate, and isovalerate but these increases did not achieve statistical significance due to variability in replicate cultures. Propionate-supplemented cultures decreased the relative abundance of Bacteroidetes (*P* = 0.03). Lactate isovalerate, isobutyrate, and succinate-supplemented cultures did not result in any statistically significant changes at the phylum level.

Analysis of community profiles at the family level resulted in the identification of a number of coherent changes induced by SCFAs, suggesting that groups of related taxa are altered in their representation by one or more SCFAs. Among the dominant families, butyrate-supplemented cultures resulted in the expansion of Enterobacteriaceae (20 to 41%) compared to control cultures (*P* = 0.03). Isobutyrate and isovalerate also expanded this family but did not reach statistical significance (Fig. 2b). These changes were driven primarily by the increased representation of *E. coli*. A number of SCFAs including isobutyrate (15 to 32%; *P* = 0.03), isovalerate to 27%; (*P* = 0.03), lactate to 21% (*P* = 0.03), propionate to 33% (*P* = 0.03), and valerate to 30% (*P* = 0.03) increased the relative abundance of Lachnospiraceae. These changes were driven primarily by *Blautia* spp. and others. Compared to control cultures, succinate (*P* = 0.03) and isobutyrate (*P* = 0.03) significantly increased the relative abundance of Acidaminococcaceae, primarily driven by altered relative abundance of *Acidaminococcus intestini*. Formate-supplemented cultures increased this family more modestly but was also statistically significant (*P* = 0.03). Most SCFAs reduced the relative abundance of taxa belonging to an unclassified *Flavonifractor* group, attributed to the reduced relative abundance of *Flavonifractor plautii*. Neither formate nor lactate impacted this group. All SCFAs reduced the average relative abundance of Bacteroidaceae, resulting from a number of *Bacteroides* spp.; however only butyrate, isobutyrate, and propionate-supplemented cultures achieved statistical significance (*P* = 0.03). Butyrate (*P* = 0.03), isobutyrate (*P* = 0.03), and valerate supplementation (*P* = 0.03) led to the virtual elimination of Peptostreptococcaceae, whereas acetate, isovalerate, and propionate reduced the relative abundance of this family but did not achieve statistical significance. These reductions reflected the reduced average relative abundance of *Romboutsia sedimentorum*. Ruminococcaceae was reduced in most cultures supplemented with SCFA, particularly isobutyrate (*P* = 0.03), isovalerate (*P* = 0.03), succinate (*P* = 0.03), and valerate (*P* = 0.03) due to reductions in multiple taxa, with the exception of acetate, butyrate, and formate where it was reduced more modestly.

Among the moderately abundant families, we noted SCFA-driven changes in Sutterellaceae, as the result of *Sutterella wadsworthensis*, Enterococcaceae, resulting from changes in the relative abundance of *E. faecalis*, Selenomonadaceae, and Erysipelotrichaceae, driven by the altered relative abundance of *Erysipelatoclostridium ramosum*, Clostridiaceae, resulting from contraction of a number of *Clostridium* spp. and *Emergencia timonensis*, and Veillonellaceae due to the expansion of phylotypes belonging to *Phascolarctobacterium faecium* and *Veilonella* spp. (*P* = 0.03). Additional significant changes were also noted in families of lower abundance (Fig. 2c).

### Species-Level Alterations by SCFAs

In total, we identified 66 phylotype groups that displayed statistically significant changes in relative abundance in response to one or more SCFAs. Among these cases, we observed more than two times as many instances of reduced (133) relative abundance compared to increased (65) relative abundance (Supplementary Table S3). Among the abundant taxa, *Acidaminococcus intestini* was increased in acetate-supplemented cultures (0.05 to 1.0%), butyrate (3.4%), and very strongly in valerate-supplemented cultures (13%). The relative abundance of *Anaerostipes hadrus* was elevated in cultures supplemented with lactate and succinate (3.1 to 8.7% and 5.6%, respectively) and significantly decreased by isobutyrate (0.0%), isovalerate (0.001%), and propionate (0.1%). A phylotype with similarity to *Blautia hansenii* and *B. producta* was not detected in control cultures but its relative abundance increased to 4.6% and 13.1%, in cultures supplemented with formate and isobutyrate, respectively. Another species group with similarity to *Blautia obeum* and *B. wexlerae*, present in control cultures at a relative abundance of 0.3%, was increased by several SCFAs, including acetate (1.3%), isobutyrate, (8.7%), isovalerate (4.7%), propionate (8.2%), and valerate (1.2%). Species groups that were below the limit of detection in control cultures were positively influenced by one or more SCFAs, including a separate species group with similarity to *Blautia obeum* was increased by isobutyrate (0.45%), propionate (1.0%), and valerate (1.68%), whereas another with similarity to *B. luti* and *B. massiliensis* were increased in propionate-supplemented cultures (5.83%).

The pathobiont, *Citrobacter freundii*, and its relative *C. murliniae* was present in high and moderate relative abundance in control cultures (2.1% and 0.7%, respectively) and further increased in cultures supplemented with propionate (8.9% and 2.9%, respectively). Both of these species groups were reduced in relative abundance by acetate (0.9% and 0.3%, respectively). *Dorea longicatena* was positively modulated by a number of SCFAs, including acetate (0.06 to 0.5%), butyrate (7.6%), isobutyrate (3.5%), isovalerate (4.7%), propionate (7.8%), and valerate (6.9%). *Phascolarctobacterium faecium* present in high abundance in control cultures (8.7%) was further increased by formate (12.4%), isobutyrate (12.8%), and succinate (31.9%) and decreased by valerate (5.5%). The pathobiont *Enterococcus faecalis* was increased to 1.77% in butyrate-supplemented cultures. The relative abundance of *Megamonas funiformis* was elevated by butyrate (3.42%) and valerate (0.06%). Finally, *Mitsuokella jalaundini* was increased by butyrate (3.44%). Among the 10 *Bacteroides* spp. significantly altered by SCFAs, the majority displayed reduced relative abundance (30 instances) compared to increased relative abundance (3 instances). Six *Clostridium* spp. displayed similar sensitivity to SCFAs, resulting in reduced relative abundance (21 instances) compared to increased relative abundance (3 instances; Supplementary Table S3). Based on our results it appears that taxa responsive to any SCFA are more likely to be impacted by others as the 66 phylotype groups displaying significant change did so in response to an average of 3 SCFAs of the 9 tested.

### SCFAs Modulate Fecal Communities in a Strain-Dependent Manner

Analysis of SCFA-supplemented fecal communities revealed that alterations of closely related phylotypes displayed coherent changes; however, exceptions were also noted suggesting strain-dependent differences in SCFA utilization capacity and/or fitness alterations.

Species groups comprising a large number of related strains revealed a number of instances wherein clear trends in relative abundance in response to SCFA supplementation were noted. For example, we identified 18 phylotypes with close relationship to *Blautia wexlerae*, a H_2_-consuming microbe that plays a role enabling H_2_-producing fermentation to proceed with continued efficiency. Among the 18 phylotypes, 100% displayed increased relative abundance (>fivefold compared to control cultures) in response to isovalerate supplementation, whereas 89% were increased in response to propionate and 78% and 61% increased in response to isobutyrate and butyrate, respectively (Supplementary Table S1). We identified 10 phylotypes with close relationships to *Eubacterium desmolans*. All 10 phylotypes displayed reduced relative abundance in cultures supplemented with isovalerate and propionate, whereas 90% and 80% displayed reduced relative abundance in cultures supplemented with succinate and lactate, respectively. The remaining phylotypes defining this species group remained unchanged.

Finally, we identified 4 phylotypes with close similarity to *Sutterella wadsworthensis*. Cultures supplemented with butyrate, acetate, isobutyrate, isovalerate, and propionate promoted the increased relative abundance of these related phylotypes. The coherence of these changes is conspicuous but not universal as some species groups displayed more heterogeneous responses. For example, 6 phylotypes defining a species group related to *Intestinibacillus massiliensis* displayed coherent responses (reduced relative abundance) to butyrate, isobutyrate, and isovalerate, whereas in response to succinate, 3 phylotypes were increased and 3 phylotypes were decreased. Taken together, we conclude that SCFA-induced fitness alterations are strain-dependent.

### Genome Reconstruction of SCFA Synthesis Pathways in Fecal Communities

**Fig. 3:**
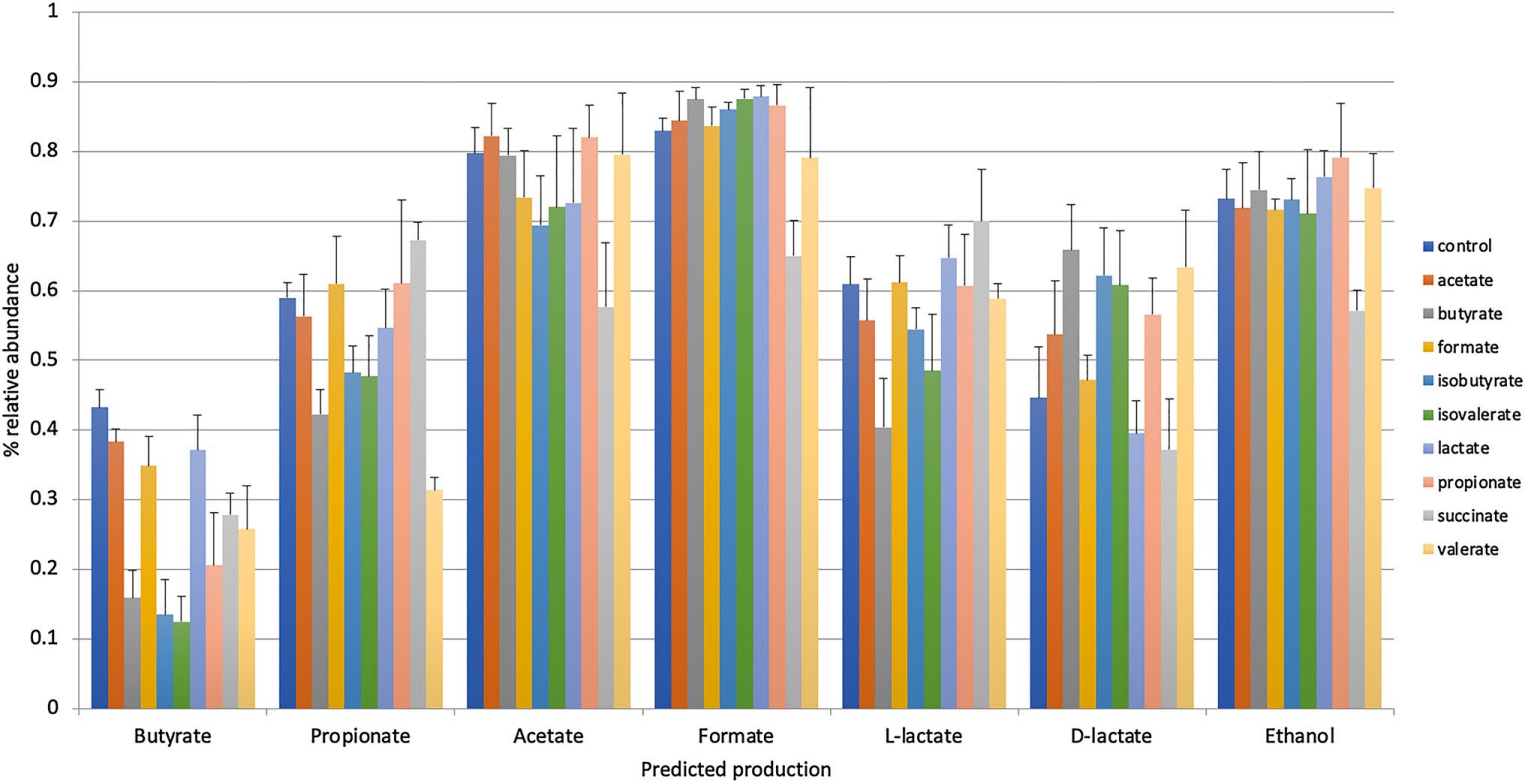
SCFA supplementation alters the representation of SCFA biosynthetic capacity. The relative abundance of communities (y-axis) predicted capacity to synthesize SCFAs and products of fermentation (x-axis) are depicted for each tested SCFA-supplemented culture (colored bars) (Color figure online)

We used genome reconstruction of reference genomes pertaining to over 600 gut-associated microbes to predict metabolic phenotypes to calculate the sample-by-sample Community Phenotype Indices (CPIs) for production pathways for four SCFAs and two isoforms of lactate. Compared to control cultures, all SCFA-supplemented cultures (except formate) displayed a reduction in butyrate producers (*P* = 0.03), and this was particularly evident in cultures supplemented with butyrate, isobutyrate, isovalerate, and propionate (Fig. 3; Supplementary Table S4). The relative abundance of predicted propionate producers was significantly reduced by butyrate, isobutyrate, valerate, and isovalerate (*P* = 0.03). The relative abundance of acetate producers was largely unaffected by SCFA supplementation with the exception that succinate-supplemented cultures displayed a substantial reduction (*P* = 0.03). The profile observed for succinate supplementation was also evident for predicted formate and ethanol producers (*P* = 0.03). d-Lactate-supplemented cultures also decreased the predicted abundance of formate producers (*P* = 0.03). The impact of SCFA supplementation on the relative abundance of microbes predicted to produce l- and d-lactate were distinct, butyrate-supplemented cultures reduced the relative abundance of l-lactate producers (*P* = 0.03), whereas it increased the abundance of d-lactate producers (*P* = 0.03). This pattern was noted for isobutyrate (ns) and isovalerate also (*P* = 0.03 for l-lactate only). d-lactate supplementation reduced the relative abundance of d-lactate producers but had little effect on the relative abundance of l-lactate producers. This pattern was also evident in succinate-supplemented cultures.

### SCFA Impacts B Vitamin Biosynthesis Potential

**Fig. 4:**
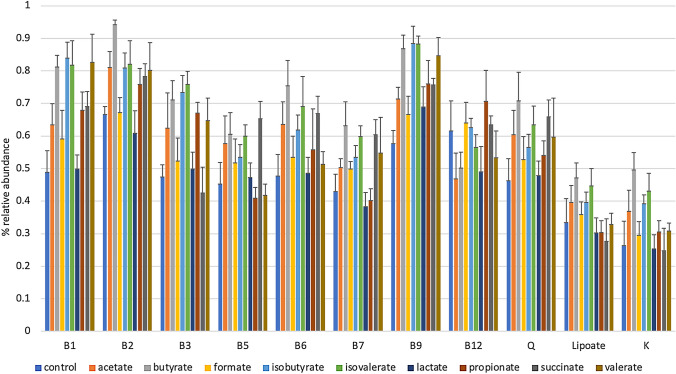
SCFA supplementation alters the representation of vitamin biosynthetic capacity. The relative abundance of communities (y-axis) predicted capacity to synthesize vitamins (x-axis) are depicted for each tested SCFA-supplemented culture (colored bars) (Color figure online)

We used the same predictive bioinformatics approach of calculate CPIs for microbial vitamin synthesis pathways. Compared to control cultures, multiple SCFA-supplemented cultures altered the abundance of B vitamin producing bacteria or prototrophs (Fig. 4; Supplementary Table S5). Acetate supplementation increased the predicted abundance of B1, B2, B3, B6, B9, and Q producers but decreased the relative abundance of B12 prototrophs. Only B2 and B9 were statistically significant (*P* = 0.03). Exogenous butyrate, isobutyrate, and isovalerate increased the relative abundance of B vitamin prototrophs, including B1, B2, B3, B6, B7, and B9 (*P* = 0.03), whereas butyrate and isovalerate increased vitamin K and Q (*P* = 0.03). Formate and lactate had little effect on any vitamin prototroph representation, only increasing the relative abundance of vitamin B9 prototrophy (*P* = 0.03). Propionate increased the relative abundance of B1, B2, B3, B9, and Q prototrophs (*P* = 0.03). Succinate increased the relative abundance of B1, B2, B5, B6, B7, and B9 prototrophs (*P* = 0.03). Finally, valerate increased the relative abundance of B1, B2, B3, and B9 prototrophs (*P* = 0.03).

### Amino Acid Biosynthesis and Degradation

According to genomic reconstructions, the majority of gut microbes are prototrophic for amino acid biosynthesis. Amino acid degradation pathways are less well conserved. These results are too complex to present in detail (Supplemental Table S6); however, certain trends are worth noting. Overall, SCFA supplementation increased the representation of predicted amino acid biosynthetic potential for all amino acids, many achieving statistical significance, whereas others did not. Lactate supplementation represents a contrast to this general trend, resulting in reductions of these pathways, except for cysteine and asparagine biosynthesis pathways. Only isoleucine, valine, leucine, and serine achieved statistical significance. Formate and succinate supplementation had the smallest effect on these pathways, except that succinate increased tryptophan biosynthesis pathway representation (*P* = 0.03) and strongly decreased asparagine representation (*P* = 0.03).

**Fig. 5:**
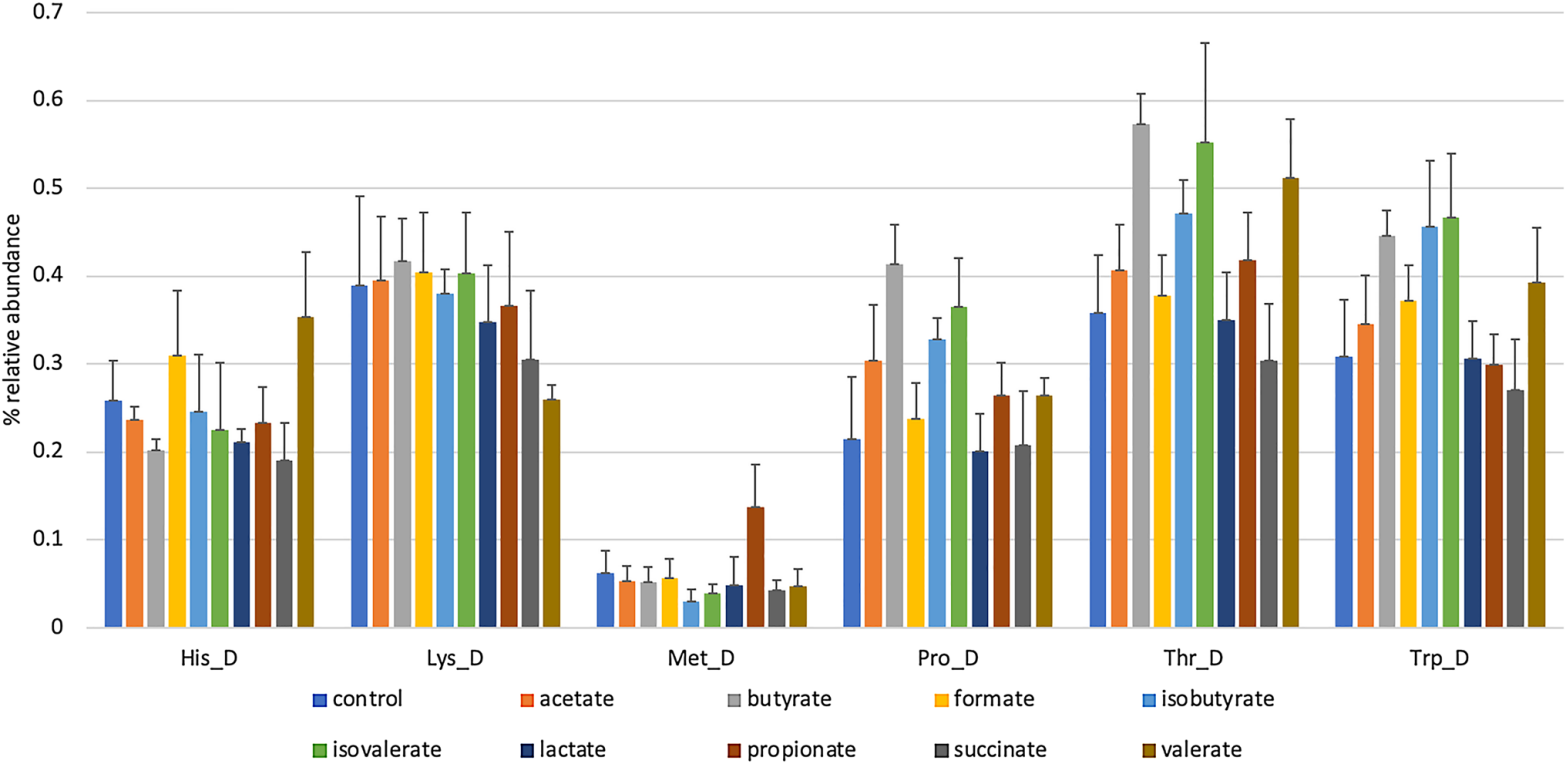
SCFA supplementation alters the representation of amino acid degradation pathways. The relative abundance of communities (y-axis) predicted capacity to degrade amino acids (x-axis) are depicted for each tested SCFA-supplemented culture (colored bars) (Color figure online)

We also analyzed the impact of SCFA supplementation on amino acid degradation pathways. These pathways are relevant as the products of these pathways, for example, tryptophan degradation products act as signaling molecules impacting the host [17–19]. These products are in some instances linked to SCFA production. The degradation of different amino acids has different fates, most funneling into central metabolism. Proline and Histidine are degraded to glutamate. Methionine and Threonine are degraded to 2-Oxobutanoate, which may then be converted to propionate. Branched chain amino acids such as valine and isoleucine are also fermented to propionate. Lysine is degraded to glutarate or crotonoyl-CoA, which may then be converted to butyrate. Tryptophan can be degraded by several microbial pathways/enzymes that produce indole, tryptamine, indoleacetate, indolepropionate, or may be fully degraded to Acetyl-CoA. Butyrate had the largest impact on degradation pathways (Fig. 5; Supplementary Table S7). These effects were mixed, increasing proline, threonine, and tryptophan degradation pathway representation (*P* = 0.03) and decreasing histidine degradation pathway abundance. Valerate-supplemented cultures decreased lysine degradation potential. Isovalerate increased the representation of microbes capable of degrading threonine (*P* = 0.03).

## Discussion

Recently, the gut microbiota has come into sharp focus as a central intermediary between diet and human health. Many studies have evaluated the modulatory effects of various prebiotic fibers that promote the increased production of SCFAs [20, 21]. Numerous prebiotics promote blooms of *Bifidobacterium* spp. that increase the amount of lactate in the colon [22, 23], whereas other prebiotics enhance the production of butyrate and/or propionate that have been studied for their effects on human health [24, 25]. Butyrate is known to have significant effects in maintaining gut homeostasis [26]. Butyrate is the primary energy source of differentiated intestinal epithelial cells where its metabolism serves to consume oxygen, thereby maintaining an anaerobic environment that maintains the fitness of the strict anaerobes populating the gut [27]. Butyrate is also known to act as an HDAC inhibitor manifesting pleiotropic alterations in transcription and host physiology [28, 29] and exerting an influence on the activation of anti-inflammatory Treg cells [30, 31]. Animal and human data demonstrated that acetate beneficially affects host energy and substrate metabolism via secretion of gut hormones like glucagon-like peptide-1 and peptide YY which affects appetite, via a reduction in whole-body lipolysis, systemic pro-inflammatory cytokine levels, and via an increase in energy expenditure and fat oxidation [32].

Despite numerous studies indicating the importance of microbially produced SCFAs on human health, little is known about how SCFAs produced by gut bacteria participate in cross-feeding to influence community structure and function. Herein, we employed in vitro anaerobic cultivation of fecal microbiota to evaluate the impact of SCFAs as potential modulators of gut communities. We speculated that the SCFAs produced by gut microbes may be consumed by others, thereby altering their fitness and relative abundance in microbial communities. We profiled fecal communities by 16S rRNA gene sequencing and identified numerous taxa that were positively or negatively influenced by cultivation in the presence of single-SCFA substrates.

### SCFAs Alter Community Structure of Fecal Microbiomes

This study demonstrates the capacity of SCFAs to modulate fecal communities at multiple taxonomic levels. High-resolution analysis of 16S rRNA sequences allowed the identification of 66 species groups that displayed increased/decreased relative abundance as the result of media supplementation with a single SCFA compared to control cultures. Based on our results, SCFA consumption or fitness alterations are rarely evident for all phylotypes belonging to a species group, which suggests that SCFA-induced alteration in fitness is a strain-dependent phenotype. We noted numerous examples wherein the majority of phylotypes belonging to a species group responded positively or negatively to one or more SCFAs (Supplementary Tables S2 and S3). For example, *Ruminococcus obeum* was positively impacted by acetate, propionate, and valerate. The relative abundance of multiple *Blautia* spp. including *B. luti*,* B. marasmi*,* B. producta*, and *B. wexlerae* were increased by multiple SCFAs as were *Dorea longicatena*. Some phylotype groups such as *Oscillibacter* spp. and *Bacteroides* spp. were negatively impacted by multiple SCFAs. An explanation for the negative response of some taxa remains unclear. Small but statistically significant reductions in relative abundance of taxa may reflect modulations induced by increases of other taxa. Taxa exhibiting larger negative effects in response to SCFA supplementation may instead highlight phylotypes with an inherent sensitivity to SCFAs that inhibit growth by as yet unknown mechanisms.

We noted that several genera displaying broad positive responses to multiple SCFAs were assacharolytic microbes, including *Acidaminococcus*,* Sutterella*,* Phascolarctobacterium*,* Veillonella*, and *Alistipes*. We speculate that these organisms may have evolved mechanisms to utilize SCFAs for energy given the inability to generate energy from sugar fermentation.

There are relatively few studies that examined cross-feeding of SCFAs, although some reports do serve to validate our findings. While a number of gut microbes generate lactate, lactate is not observed in the colon at appreciable concentrations which suggests that it is consumed by microbes [33]. This hypothesis was tested in a study to identify lactate consumers. When the lactate producing *Bifidobacterium adolescentis* was co-cultured with *Anaerostipes caccae* or *Eubacterium hallii* in the presence of starch, lactate levels remained low due to consumption by *A. caccae* and *E. hallii* by converting consumed lactate to butyrate [33, 34]. Indeed, most *Anaerostipes* spp. consume both lactate and acetate [35]. In the current study, we did not observe *E. hallii*; however, *Anaerostipes hadrus* was profiled and was positively modulated by lactate. Among the 21 phylotypes mapping to the *Anaerostipes hadrus* species cluster, nine displayed >fivefold increased relative abundance in acetate-supplemented cultures, whereas 19 displayed >twofold increased relative abundance. Lactate-supplemented cultures increased the relative abundance of 4 phylotypes >fivefold and 14 phylotypes >twofold relative abundance. Nearly identical profiles were observed for cultures supplemented with succinate.

It has been known for some time that in the oral cavity, where lactate is the principle microbial product promoting dental caries, that *Veillonella* spp. consume and therefore remediate the acidifying effects of lactate produced by *Streptococcus* spp., following sugar consumption [36, 37]. In our profiling, we identified 2 phylotypes related to *Veillonella atypica*. Both of these phylotypes displayed increased relative abundance >30-fold in lactate-supplemented cultures. One of these also displayed increased relative abundance in acetate and valerate-supplemented cultures.

Co-culture experiments in media containing xylan, with the succinate-producing *Paraprevotella xylaniphila*, resulted in the increased growth of *P Phascolarctobacterium faecium* by 2–3 orders of magnitude [38]. Propionate was generated as the result of succinate consumption via succinate decarboxylation reactions. We observed 12 phylotypes related to *P. faecium*, nine of which displayed >fivefold increased relative abundance in succinate-supplemented cultures, all 12 displayed >2.5-fold increases relative to control cultures. The related genus *Acidaminococcus* is also a succinate consumer. Our results indicated that two phylotypes related to *A. intestini* displayed increased relative abundance of ~2 and eightfold in succinate-supplemented cultures. It is notable that the relative abundance of these phylotypes were more robustly increased in cultures supplemented with butyrate, acetate, and valerate. Utilization of these substrates has not been reported previously.

### SCFAs Alter Community Metabolism

To assess the functional implications of SCFA-driven changes in microbiota composition, we applied genome reconstruction of metabolic pathways present in profiled microbes for which one or more complete reference genomes were available. The impact of SCFA supplementation on the relative abundance of the predicted community members possessing complete SCFA production pathways (CPIs) was assessed (Fig. 3). All predicted pathway representation were impacted by at least one SCFA. The strongest effects were noted in cultures supplemented with butyrate that significantly reduced the predicted CPIs of several SCFA production pathways. By contrast, most SCFA supplementation had little effect on predicted formate, acetate, and ethanol producers. Predicted producers of l- and d-lactate in SCFA-supplemented cultures were similar in some instances but more often were disparate. For example, butyrate, isobutyrate, and isovalerate supplementation resulted in reduced predicted l-lactate producers but increased d-lactate production. Conversely, succinate-supplemented cultures resulted in increased predicted l-lactate but decreased predicted d-lactate production. It remains unclear whether these observations reflect happenstance changes based on fitness profiles or if instead they highlight regulatory circuits based on positive and/or negative feedback control.

Analysis of reconstructed pathways for B vitamin biosynthesis, including lipoic acid and vitamins K and Q, further illustrate the functional implications of SCFA-driven restructuring of fecal communities (Fig. 4). Surprisingly, most SCFA-supplemented cultures generated communities predicted to generate these metabolites were increased or remained similar compared to control cultures. Interestingly, lactate supplementation generally resulted in reduction of vitamin biosynthetic potential, with the exception of vitamin B9. It should be noted that some but not all of the increases in vitamin production pathways were driven primarily by increased representation of dominant, multi-prototrophs, such as *E. coli*.

Finally, we analyzed the impact of SCFA supplementation on community’s metabolic potential for amino acid biosynthesis (Supplementary Table S7) and degradation (Fig. 5). Alterations in multiple amino acid prototroph representation were noted. Perhaps of greater consequence to host physiology, SCFA-induced changes in amino acid degradation pathways were noted. With the exception of tryptophan, products of amino acid degradation are not well studied. Tryptophan and its degradation products are sensed by the host through aryl hydrocarbon receptors that alter cell signaling resulting in local and systemic effects.

The study of prebiotics is advancing rapidly based on the large body of data showing that prebiotics alter gut communities and the amount of SCFAs produced. Given, the potential clinical utility of controlling microbial-produced SCFAs levels through prebiotic interventions, little has been elucidated regarding SCFA consumption in gut communities. Ultimately, SCFA levels represents an equilibrium dictated by microbial SCFA production, host absorption, and microbial consumption. Our in vitro study is both a strength and a limitation. By analyzing microbial fitness in response to SCFAs in vitro, we effectively remove host complexities such as absorption that may prevent the identification of SCFA consumers in microbial communities. It remains unclear how the results presented here will translate to in vivo studies. A second limitation of the current study is the inability to attribute the effects of SCFA supplementation as a direct or indirect effect on gut microbiota community structure. It is conceivable that direct effects involving taxa that utilize one or more SCFAs with commensurate fitness gain are relatively innumerous and that the majority of the observed changes in phylotype abundance were driven by indirect effects, mediated by cross-feeding of unknown metabolites or inhibitors produced by taxa directly impacted by treatment.

In summary, we identified 66 distinct phylotype groups in cultures supplemented with one or more SCFAs displaying altered fitness as determined by statistically significant changes in relative abundance (Supplementary Table S3). The improved fitness of these species suggest that these fitness changes are driven by the consumption of SCFAs to generate energy. We cannot rule out the possibility that increased fitness may reflect indirect effects. Additional studies are required to verify whether these taxa indeed consume SCFAs and derive fitness advantage as a result. We conclude that SCFA-driven fitness is a strain-dependent phenomenon. This suggests that experimental validation of our predictions may require the isolation of multiple strains pertaining to species of interest. A growing number of laboratories have enhanced gut microbiome isolate banks and therefore are well poised to test candidate SCFA consumers in future work.

## Supplementary Information

Below is the link to the electronic supplementary material.

Supplementary file1 (XLSX 212 kb)

Supplementary file2 (DOCX 15 kb)

## Data Availability

All 16S rRNA sequences have been deposited at NCBI SRA database and can be found at https://www.ncbi.nlm.nih.gov/genbank/. The Bioproject ID is PRJNA725898 and accession numbers are SAMN18914736–SAMN18914775.
