# Supplementary material for: Short-Chain Fatty Acids Modulate Healthy Gut Microbiota Composition and Functional Potential
Source: Curr Microbiol. 2022 Mar 14;79(5):128. doi: 10.1007/s00284-022-02825-5 (PMC8921067; doi:10.1007/s00284-022-02825-5)
Supplement: Supplementary file 2 — Supplementary file2 (DOCX 15 kb) [file 284_2022_2825_MOESM2_ESM.docx]

**Supplementary Table legends**

**Table S1. Statistical analysis of significant changes in taxa and biochemical pathway representation induced by SCFAs.**

**Table S2. Modulatory capacity of SCFAs at the phylotype level.** The average relative abundance of individual phylotypes and species groups observed in SCFA-supplemented compared to control cultures were used to determine ratios (fold-change). Phylotypes displaying increased relative abundance >5-fold (green), decreased relative abundance (<5-fold) (red) or unchanged (<5 or >0.2-fold), yellow. Taxa unobserved are shown in black. To create ratios for all phylotypes zeros were replaced by values of 1X10^-5^ to approximate the limit of detection.

**Table S3. Species groups significantly altered by one or more SCFAs.** SCFAs tested are shown in row 1. Species groups positively impacted are shown in green, species groups negatively impacted are shown in red.

**Table S4. SCFAs alter the representation of SCFA biosynthetic capacity.** The average predicted abundance (CPI) for SCFA biosynthesis and fermentation products in control and SCFA-supplemented cultures.

**Table S5. SCFAs alter the representation of vitamin biosynthetic capacity.** The average predicted abundance (CPI) for B, K and Q vitamin and lipoic acid biosynthesis in control and SCFA-supplemented cultures.

**Table S6. SCFAs alter the representation of amino acid biosynthetic capacity.** The average predicted abundance (CPI) for amino acid biosynthesis in control and SCFA-supplemented cultures.

**Table S7. SCFAs alter the representation of amino acid degradation capacity.** The average predicted abundance (CPI) for amino acid degradation in control and SCFA-supplemented cultures.
